# Characterizing the repeatability of cardiovascular responses to hypoxic apneas in adults

**DOI:** 10.14814/phy2.70692

**Published:** 2025-12-19

**Authors:** Desmond A. Young, Benjamin R. O'Croinin, Mackenzie I. Farris, Sean van Diepen, Craig D. Steinback

**Affiliations:** ^1^ Neurovascular Health Lab, Faculty of Kinesiology, Sport, and Recreation University of Alberta Edmonton Alberta Canada; ^2^ Department of Critical Care Medicine, Faculty of Medicine and Dentistry University of Alberta Edmonton Alberta Canada; ^3^ Division of Cardiology, Department of Medicine, Faculty of Medicine and Dentistry University of Alberta Edmonton Alberta Canada

**Keywords:** arrhythmia, bradycardia, breath‐hold, heart rate, reliability

## Abstract

Apneas performed during hypoxia cause pronounced bradycardia and cardiac arrhythmias. What remains unclear is the intraindividual consistency of these responses. Therefore, we assessed the within‐day and between‐day repeatability of responses to hypoxic apneas, including the genesis of arrhythmias or electrocardiogram (ECG) changes. Sixteen participants (8F/8M, 25 ± 3 years, BMI 23.7 ± 3.4 kg/m^2^, mean ± SD) completed four hypoxic apneas per day on consecutive days, time‐matched to a maximal apnea on day 1 (39 ± 16 s). We quantified heart rate (HR) and mean arterial pressure (MAP) repeatability using within‐subject standard deviations (WSSD), [95%CI]. We hypothesized that WSSDs would be <5 bpm and <4 mmHg for HR and MAP. We also assessed the repeatability of arrhythmias or ECG changes. Within‐day, the HR WSSD was 4.4 bpm [3.7, 5.6], and the MAP WSSD was 4.1 mmHg [3.4, 5.1]. Between‐day comparisons had somewhat higher WSSDs: HR was 5.7 bpm [5.0, 6.5] and MAP was 5.0 mmHg [4.4, 5.8]. Arrhythmias or ECG changes occurred during 45% of apneas. These data indicate poor repeatability of electrical conduction changes during apneas. Conversely, our WSSD data indicate good within‐day and between‐day repeatability, albeit somewhat higher than hypothesized. These data therefore indicate that hypoxic apneas are a repeatable laboratory stressor for HR and MAP, but that researchers should exercise caution in interpreting individual‐level arrhythmia data.

## Graphical abstract

Heart rate and mean arterial pressure showed robust repeatability during hypoxic apneas. Conversely, arrhythmias occurred inconsistently, indicating non‐repeatable mechanisms triggering arrhythmias.

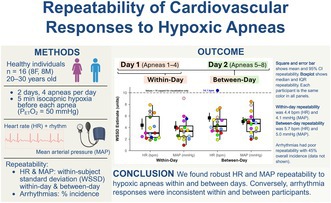

## 1 INTRODUCTION

Recent investigations have consistently demonstrated that decreased inhaled oxygen content (i.e., hypoxia) before a voluntary apnea is associated with a profound bradycardic response during the apnea (Busch et al., 2018, 2020). This is an oxygen‐specific response, augmented by hypoxia compared to normoxia, and not affected by modulations in inhaled carbon dioxide (O'Croinin et al., 2024). Chronic high‐altitude hypoxic exposure (i.e., days) appears to further potentiate the bradycardic response to apneas and increases the incidence of cardiac arrhythmias during apneas compared to acute exposure of minutes to hours (Busch et al., 2021). In fact, a longitudinal study suggested that increased arrhythmogenesis during high‐altitude apneas occurs after several days at altitude secondary to chemoreflex sensitization (Berthelsen et al., 2022). Despite these findings, no study has assessed the intra‐individual repeatability of cardiac responses to hypoxic apneas. For example, it remains unclear whether arrhythmias—or the lack thereof—during an apnea are a characteristic of an individual or transient and random. Given the potential research interest in arrhythmias during apneas (Mulder et al., 2023), it is crucial to understand the consistency with which these cardiac phenomena occur in an individual.

Beyond the responses to the apneas themselves, their transient nature makes data analysis challenging. In addition, different research groups have used varying analysis methods to quantify cardiovascular responses to apneas (see Mulder et al. (2023) and O'Croinin et al. (2024) for two approaches), making direct comparisons between studies more challenging. Our group has previously quantified apnea responses as the longest RR interval for heart rate, or the beat with the highest mean arterial pressure, in the final 10 cardiac cycles of an apnea (Berthelsen et al., 2022; Busch et al., 2018, 2020, 2021; O'Croinin et al., 2024). However, a single beat may not capture a repeatable response. As such, we devised three alternative methods to assess apnea heart rate and mean arterial pressure (see Section 2). We then compared the methods to determine the effect of data analysis on estimates of intraindividual repeatability.

The purpose of this study was to quantify the intraindividual repeatability of cardiovascular responses to hypoxic apneas. We hypothesized that within‐day repeatability comparisons would indicate a within‐subject standard deviation (WSSD) < 5 bpm for heart rate and WSSD < 4 mmHg for mean arterial pressure when assessed as absolute changes from free‐breathing to the end of the apnea. We further hypothesized that between‐day comparisons would increase the WSSD, and that inconsistency between apneas would primarily stem from random variability. These WSSD estimates were derived from a cohort study that found 5 bpm and 4 mmHg WSSD at rest across two days of measurements in 822 individuals (Stanforth et al., 2000). Other studies examining repeatability during isometric handgrip, postexercise circulatory occlusion, cold pressor test, and cycle ergometer exercise have also noted similar repeatability (Dillon et al., 2020; Wergel‐Kolmert et al., 2002).

## 2 MATERIALS AND METHODS

### 2.1 Study design and participants

This study used a prospective within‐subjects design. Nonsmoking males and females were recruited using non‐probability quota sampling. Exclusion criteria were ages below 18 or above 70 or known cardiovascular or nervous system disease, as assessed by a questionnaire. Resting blood pressure was also > 90/60 and < 139/85 mmHg for all participants. Female participants self‐reported that they were not pregnant at the time of data collection. The phase of the ovarian cycle was not standardized but was recorded. The study was approved by the University of Alberta Health Research Ethics Board (Pro00138947) and conformed with the standards of the latest *Declaration of Helsinki* including registration in a public database (ClinicalTrials.gov NCT06399575). All participants provided written informed consent prior to participating. Seventeen participants were recruited for the study. One participant withdrew on day 1 due to discomfort with the hypoxic exposure, resulting in a final sample size of 16.

### 2.2 Protocol and procedures

All testing sessions took place at the University of Alberta (676 m elevation). Participants underwent two in‐lab testing sessions beginning at the same time of day on consecutive days. Participants arrived at the laboratory after fasting for a minimum of 2 h; they also abstained from caffeine, alcohol, and strenuous physical activity for at least 12 h before each visit. After participants provided informed consent, they completed a slow vital capacity test in triplicate to measure lung volume; this was used to standardize the amount of air in their lungs during each apnea.

**FIGURE 1:**
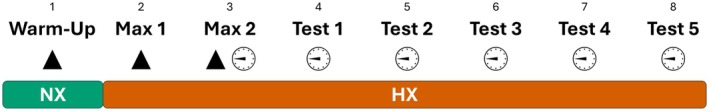
Characteristics of the eight apneas per testing day. The warm‐up apnea was performed during normoxia (NX), and each subsequent apnea was performed after a 5‐min hypoxic period (HX). The triangles (▲) indicate the apneas with a maximal effort; all other apneas were held to a target duration (see text for details). The timers (⏲) indicate the apneas where participants saw their apnea duration in real time.

Each test began with a 10‐min baseline period where participants breathed through a mouthpiece to determine resting values for heart rate, mean arterial pressure, and end‐tidal partial pressures of oxygen and carbon dioxide. Participants then performed eight apneas (Figure 1). Their first apnea was a warm‐up for familiarization, though participants were encouraged to perform an all‐out effort. The remaining seven apneas each followed a 5‐min hypoxic period. To achieve hypoxia, a dynamic end‐tidal forcing system decreased the end‐tidal partial pressure of oxygen to 50 mmHg, corresponding to 80%–85% peripheral oxygen saturation, and maintained end‐tidal carbon dioxide at baseline levels. The first hypoxic apnea was a maximal effort where participants did not see their apnea duration in real time. Participants were then told the duration of this apnea, and performed a second maximal hypoxic apnea, this time with a live view of their apnea duration. The duration of the longest of the two maximal hypoxic apneas was used as the target duration for the five remaining test apneas. Participants had a live view of their apnea duration for all five test apneas and were instructed to hold their breath for the target duration, but no longer, to their best ability. Throughout the protocol participants had at least 5 min of rest between the end of an apnea and the beginning of the next hypoxic period. The rest was extended as needed to allow heart rate and mean arterial pressure to return to within 5 units of their baseline values. Immediately following each apnea, participants rated the difficulty of the apnea using a 10‐point modified Borg Scale (Foster et al., 2001).

On day 2 participants completed the same protocol except for the slow vital capacity test. The day 2 maximal hypoxic apneas were used only to provide a similar “warm‐up” before the test apneas and as a measure of learning from day 1. The test apneas on day 2 had the same target duration as day 1. On day 2 the end‐tidal partial pressure of carbon dioxide was also set to the baseline value from day 1 which eliminated any differential effects of carbon dioxide on between‐day repeatability.

Throughout the protocol we standardized lung volume during apneas. On the final breath before each apnea a researcher instructed participants to exhale fully. The researcher then turned a three‐way valve to a 3 L calibration syringe (Model 5530, Hans Rudolph) prefilled with the prevailing hypoxic gas mixture from the dynamic end‐tidal forcing system to 40% of the participant's vital capacity. After inhaling this volume, the participant immediately began their apnea. For warm‐up apneas the syringe was loaded with room air.

### 2.3 Measures

Participants were instrumented with an electrocardiogram (ECG; lead II) for heart rate and rhythm measurements and a finger photoplethysmography cuff on the right middle finger for continuous blood pressure measurements (Finometer Pro, Finapres Medical Systems). Finger pressure was calibrated to manual blood pressure measurements taken in triplicate on the left arm during baseline. A finger pulse oximeter on participants' right index finger measured peripheral oxygen saturation (Nellcor N‐600x, Medtronics). Finally, participants wore a respiratory belt with a strain gauge (Model TN1132/ST, ADInstruments) that measured chest circumference to assist in detecting breathing movements and the end of each apnea.

Participants wore a nose clip and breathed through a mouthpiece with a sampling port connected to oxygen (S‐3A/I Oxygen Analyzer & Model N‐22M Sensor, AEI Technologies) and carbon dioxide (CD‐3A Carbon Dioxide Analyzer & P‐61B Sensor, AEI Technologies) gas analyzers run in parallel. The dynamic end‐tidal forcing system manipulated inspired fractions of oxygen, carbon dioxide, and nitrogen based on the discrepancy between actual and target end‐tidal oxygen and carbon dioxide using custom software (LabVIEW 16.0, National Instruments). Inspired fractions and volumes were adjusted breath‐by‐breath to achieve the target isocapnic hypoxia stimulus.

### 2.4 Sample size calculation

For this investigation, the sample size was determined based on the uncertainty of the WSSD estimate relative to the population parameter using the following equation (McAlinden et al., 2015):

$$z_{1-\alpha /2}\frac{\text{WSSD}}{\sqrt{2n\left(m-1\right)}}=\text{WSSD}\times \mathrm{LC}$$

where *z* is the z‐score at a given level of confidence, *n* is the sample size, *m* is the number of repeated trials, and LC is the desired level of uncertainty between the sample WSSD and the population parameter. Using 20% uncertainty between the sample estimate and the population parameter (LC = 0.20), a 95% confidence interval (*z* = 1.96), and four repeated trials (*m* = 4), we required 16 participants. Of the five test apneas per day, four were included in analyses. We decided a priori to use the first four apneas and discard the final one unless an apnea duration deviated more than 2 s from the target time, or if the heart rhythm was uninterpretable during analysis. In addition, we decided a priori to discard the same apnea from both days when possible.

### 2.5 Data analysis

To assess intraindividual apnea responses, we quantified within‐day and between‐day repeatability. For the purposes of hypothesis testing, data from day 1 was used for within‐day assessments, and data from both days was used for between‐day assessments. We did not conduct repeatability analyses with data from only day 2. That said, for complete transparency we have presented all repeatability data from day 1, day 2, and both days in Tables S1 and S2.

All data were collected at 1 kHz using LabChart software (Chart Pro, v8.1.3, ADInstruments). Mean arterial pressure was calculated by averaging the blood pressure waveform over each cardiac cycle. Minute ventilation was calculated as the product of breathing frequency and tidal volume; the latter was determined as the integral of the inspired portion of the respiratory pneumotach trace. Data were then extracted into a spreadsheet for analysis.

Baseline data were used solely to establish a benchmark to assess recovery within a testing session and were not used for analytical purposes. Free‐breathing values were extracted as one‐minute averages 120–60 s before each apnea to ensure that a stable free‐breathing baseline was obtained without influence from anticipation which tends to increase heart rate and minute ventilation immediately before apneas (Caspers et al., 2011; Schagatay et al., 1999). Apnea data were extracted beat by beat.

**FIGURE 2:**
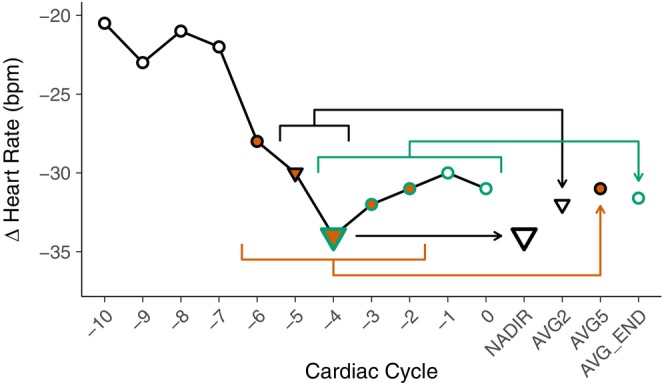
Visual representation of methods used to analyze heart rate during apneas. The nadir beat (NADIR) is enlarged. AVG2 includes the NADIR and the beat preceding it (triangles). AVG5 includes the NADIR and the four closest beats (orange). AVG_END is the average of the five final beats (green). Mean arterial pressure methods were determined in the same manner except the PEAK is the highest beat in cardiac cycles −10 to 0.

We compared four “methods” of quantifying apnea heart rate and mean arterial pressure. All methods used the same search window at the end of the apnea which included the cardiac cycle during which the apnea ended (beat 0), determined by an inhalation measured by the respiratory belt, and the 10 cardiac cycles immediately preceding it (beats −10 to −1). First, the NADIR for heart rate was determined as the single nadir beat in the search window, and the PEAK for mean arterial pressure was determined as the single highest beat in the search window (Figure 2). Next, the AVG2 method averaged the NADIR/PEAK and the beat preceding it. The AVG5 method averaged the NADIR/PEAK and the four closest beats. Finally, the AVG_END averaged the five final beats in the search window. In any instance where the NADIR/PEAK beat occurred at beat 0, −1, −9, or −10 the AVG2 and AVG5 calculations were shifted such that only beats −10 to 0 were included in any calculations. If two beats in the search window were tied for NADIR/PEAK, the beat closer to the end of the apnea was used.

The change in heart rate and mean arterial pressure during apneas was calculated as apnea minus free‐breathing. The nadir peripheral oxygen saturation was calculated as the lowest value during the apnea or within 1 min following the end of the apnea to account for the circulatory delay in detecting peripheral oxygenation; the change in peripheral oxygen saturation was calculated as nadir minus free breathing. One participant consistently desaturated below 60% for all 14 hypoxic apneas (range 26%–55%). The Nellcor N‐600x has only been validated down to 60% peripheral oxygen saturation (Covidien, 2011). Therefore, values below 60% were replaced with 60% for summary statistics to prevent inflating averages and were excluded from repeatability analyses due to an unknown degree of instrumental error. We determined apnea duration using the airflow readings from the pneumotach at the start of apnea and the chest movement measured by the respiratory belt at the end of apnea. To identify arrhythmias or ECG changes, a trained researcher (DAY) visually inspected the ECG during all apneas with a search window from the start of the apnea up to and including the third cardiac cycle after the end of the apnea. A second trained researcher (BRO) then reviewed all apneas identified as not having an arrhythmia or ECG change. Finally, a cardiologist (SvD) reviewed all suspected arrhythmias or ECG changes and confirmed their presence and classification. Sinus bradycardia was not classified as an arrhythmia due to its high prevalence during voluntary apneas.

### 2.6 Statistical analyses

Continuous data are reported as mean ± standard deviation or value [95% confidence interval (CI)]. Discrete data are reported as median and interquartile range (IQR). All statistical analyses were completed in R Statistical Software (v4.4.1 R Core Team 2024). To assess absolute repeatability, we computed within‐subject standard deviations (WSSD). The WSSD is the typical amount of error we can expect in a measurement and is calculated using a two‐way ANOVA without an interaction term (Hopkins, 2011; Weir, 2005). The participant and trial number (i.e., the identity of the apnea in a sequence of repeated measurements) were factors and the WSSD is the square root of the mean square of residuals (Hopkins, 2011; Weir, 2005). We also calculated confidence intervals for the WSSD estimates using the following formula (Hopkins, 2007; Tate & Klett, 1959):

$$\left[\sqrt{\frac{\mathrm{df}\cdot {\text{WSSD}}^{2}}{\chi_{\alpha /2,\mathrm{df}}^{2}}},\sqrt{\frac{\mathrm{df}\cdot {\text{WSSD}}^{2}}{\chi_{1-\alpha /2,\mathrm{df}}^{2}}}\right]$$

where *df* is the degrees of freedom for the residuals term in the ANOVA used to calculate the WSSD, and $\chi_{\alpha /2,\mathrm{df}}^{2}$ and $\chi_{1-\alpha /2,\mathrm{df}}^{2}$ are chi‐square values for the upper and lower tails, respectively, at a pre‐specified alpha.

We also modeled between‐participant heterogeneity in repeatability using the individual WSSD (WSSD_*i*_). WSSD_*i*_ approximates the WSSD and represents the standard deviation of the outcome variable for participant *i* across trials. WSSD_*i*_ is biased because it does not account for the trial number and is therefore affected by systematic differences in the group mean (Hopkins, 2011). WSSD_*i*_ analyses were used to identify statistical outliers, defined as WSSD_*i*_ values falling more than 2.5 × IQR above the third quartile or below the first quartile. For ease of comparison between analysis methods for heart rate and mean arterial pressure, outliers were flagged for all four methods if they met the criteria for at least one method. WSSD analyses were rerun without outliers as sensitivity analyses, though all interpretations are based on analyses with outliers included. Further details of outlier detection and sensitivity analyses are presented in Figures S1 and S2, and Table S3.

We compared the WSSD estimates between analysis methods (e.g., NADIR) using confidence interval overlap. The single group WSSD derived from the ANOVA and its asymmetrical confidence interval meant we could not compare WSSD estimates using a *t*‐test or ANOVA. In addition, the biased WSSD_*i*_ is contaminated by systematic changes in the group mean and we cannot assume that it is proportional to the WSSD estimates. An 84% confidence interval has been empirically shown to closely match two‐tailed statistical tests comparing means using an alpha of 0.05 (*p* < 0.05 for statistical significance) with even or uneven confidence intervals (MacGregor‐Fors & Payton, 2013). Any two 84% confidence intervals that do not overlap are considered different (*p* < 0.05). We only present 84% CIs for comparing methods and otherwise use 95% CIs.

To assess relative repeatability, we calculated two‐way mixed effects, absolute agreement, single measurement intraclass correlation coefficients (ICC) for heart rate, mean arterial pressure, peripheral oxygen saturation, and minute ventilation (Koo & Li, 2016). Residuals versus fits plots and density plots of residuals were used alongside the Shapiro–Wilk test and Breusch‐Pagan test to check the normality and homoscedasticity assumptions of the WSSD and ICC calculations (Blanca et al., 2017; Gaonkar & Beasley, 2023; Li et al., 2024; Shatz, 2024).

Arrhythmias and ECG changes were analyzed using descriptive statistics, including the number of unique participants with a specific arrhythmia type. We also calculated three incidence metrics. Overall incidence was defined as the percentage of apneas with an arrhythmia across all 128 test apneas (16 participants × 8 test apneas = 128). Affected individuals' incidence represents the median (IQR) intraindividual arrhythmia incidence, calculated only from participants who had at least one arrhythmia. Finally, all individuals' incidence is the median (IQR) intraindividual arrhythmia incidence, including all participants.

To quantify systematic differences in apnea responses between days (i.e., bias), we calculated the mean difference in responses on day 1 versus day 2. Bias between days was also assessed using a paired *t*‐test with data from the four test apneas on each day. Further, an effect size of standardized mean difference was calculated (i.e., Hedges *g*_*av*_) (Lakens, 2013). Because none of the analyses utilized *p* values or relied on the outcome of a null hypothesis test per se, we did not have an a priori alpha for statistical significance.

## 3 RESULTS

### 3.1 Baseline characteristics

Participant characteristics and baseline physiological data are presented in Table 1. Five of eight females were taking hormonal contraceptives at the time of testing. Only females reported taking medications: one reported iron supplements (FeraMax), two took allergy medications (Rupall 5 mg and 10 mg), and two others reported other medications (Dexilant 60 mg; Isotretinoin 20 mg). One male reported long COVID symptoms; one female reported past supraventricular tachycardia, one reported anemia, and another reported Reynaud's disease. The baseline physiological data suggest that participants had normal resting cardiopulmonary function. End‐tidal partial pressures of oxygen were close to the 50 mmHg target, confirming the presence of a hypoxic stimulus (Table 2). The hypoxic apneas elicited a pronounced bradycardia and concomitant increase in mean arterial pressure.

**TABLE 1:** Participant characteristics and baseline physiological data 120–60 s before the warm‐up apnea on day 1.

	All (*n* = 16)	Male (*n* = 8)	Female (*n* = 8)
	Mean ± SD or *n* (%)	Mean ± SD or *n* (%)	Mean ± SD or *n* (%)
Race	White: 12 (75) Latin American: 2 (13) South Asian: 1 (6) White+Indigenous: 1 (6)	White: 6 (75) Latin American: 2 (25)	White: 6 (75) South Asian: 1 (13) White+Indigenous: 1 (13)
Age (years)	25 ± 3	26 ± 3	24 ± 3
Height (cm)	173 ± 9	179 ± 6	167 ± 7
Weight (kg)	71 ± 14	80 ± 11	63 ± 13
BMI (kg/m^2^)	23.7 ± 3.4	24.7 ± 2.2	22.8 ± 4.2
VC (L)	5.18 ± 1.44	6.34 ± 0.69	4.02 ± 0.93
Apnea Target (s)	39 ± 16	42 ± 18	37 ± 14
HR (bpm)	73 ± 13	63 ± 5	84 ± 9
MAP (mmHg)	86 ± 5	85 ± 6	87 ± 5
SBP (mmHg)	112 ± 8	116 ± 6	107 ± 7
DBP (mmHg)	72 ± 6	70 ± 6	74 ± 5
SpO_2_ (%)	97.7 ± 1.2	97.2 ± 1.3	98.2 ± 1.0
V̇E (L/min)	11.5 ± 2.4	12.4 ± 2.6	10.7 ± 2.0
P_ET_O_2_ (mmHg)	90.8 ± 5.3	90.5 ± 6.8	91.0 ± 3.6
P_ET_CO_2_ (mmHg)	38.3 ± 4.0	38.9 ± 4.0	37.8 ± 4.2

Abbreviations: BMI, body mass index; DBP, diastolic blood pressure; HR, heart rate; MAP, mean arterial pressure; *n*, sample size; P_ET_CO_2_, partial pressure of end‐tidal carbon dioxide; P_ET_O_2_, partial pressure of end‐tidal oxygen; SBP, systolic blood pressure; SD, standard deviation; SpO_2_, peripheral oxygen saturation; VC, vital capacity; V̇E, minute ventilation.

**TABLE 2:** Group mean (± standard deviation) physiological data during hypoxic free‐breathing and apneas.

		Day 1		Day 2	
		Maximal apnea^a^	Test apnea average	Maximal apnea^a^	Test apnea average
Apnea duration (s)		39 ± 16	40 ± 15	48 ± 19	40 ± 15
Free‐breathing HR (bpm)		80 ± 16	78 ± 14	79 ± 12	76 ± 12
Δ HR (bpm)	NADIR	−22 ± 10	−22 ± 9	−23 ± 9	−19 ± 9
	AVG2	−16 ± 11	−15 ± 10	−17 ± 9	−12 ± 11
	AVG5	−15 ± 11	−12 ± 9	−16 ± 9	−11 ± 11
	AVG_END	−15 ± 11	−13 ± 10	−17 ± 9	−11 ± 12
Free‐breathing MAP (mmHg)		92 ± 6	92 ± 8	92 ± 6	93 ± 7
Δ MAP (mmHg)	PEAK	+27 ± 11	+27 ± 13	+27 ± 12	+27 ± 12
	AVG2	+25 ± 11	+25 ± 12	+25 ± 12	+25 ± 11
	AVG5	+23 ± 10	+23 ± 11	+24 ± 11	+23 ± 11
	AVG_END	+22 ± 10	+22 ± 10	+24 ± 11	+22 ± 11
Free‐breathing SpO_2_ (%)		87.9 ± 2.5	87.8 ± 2.4	87.9 ± 2.3	88.1 ± 2.4
Δ SpO_2_ (%)		−8.3 ± 8.6	−10.8 ± 8.5	−11.1 ± 9.1	−10.5 ± 8.4
V̇E (L/min)		20.5 ± 6.9	23.0 ± 8.3	20.8 ± 8.8	22.5 ± 7.7
P_ET_O_2_ (mmHg)		49.7 ± 0.7	49.1 ± 1.3	49.7 ± 1.0	49.1 ± 1.1
P_ET_CO_2_ (mmHg)		38.7 ± 3.9	38.8 ± 3.9	38.8 ± 3.6	38.5 ± 3.8

*Note*: All variables with a delta (Δ) represent changes in response to apneas relative to free breathing. See text for details on NADIR, PEAK, AVG2, AVG5, and AVG_END.

Abbreviations: HR, heart rate; MAP, mean arterial blood pressure; P_ET_CO_2_, partial pressure of end‐tidal carbon dioxide; P_ET_O_2_, partial pressure of end‐tidal oxygen; SpO_2_, peripheral oxygen saturation; V̇E, minute ventilation.

^a^ Maximal apnea data are compiled from each individual's longest maximal apnea on each day, irrespective of chronology.

In five instances we used data from a participant's fifth test apnea. All occurred because participants fell short of their target apnea time, and all five instances were from unique individuals (three males, two females). Four of the short apneas occurred on day 1, with two on the third and two on the fourth apnea in the series of five. The short apnea on day 2 occurred on the first test apnea. There were no instances where data from the fifth apnea were used for any other reason.

Participants' pretest maximal hypoxic apnea durations on day 1 were 39 ± 16 s. Seven participants achieved this time on their first maximal apnea, whereas nine had their longest time on the second maximal apnea. All participants but one increased their maximal time on day 2, with an average increase of 8 ± 14 s, up to 48 ± 19 s.

Participants rated the effort of each apnea immediately after the resumption of breathing. No participant reported a maximal score on their day 1 warm‐up apnea. Conversely, participants reported maximal or near‐maximal efforts on the maximal hypoxic apneas. Most participants continued to report high ratings of perceived exertion across all test apneas (median of 9 for all except day 1 test 4 with a median of 9.5). Four individuals did not report a 10 across any of the maximal apneas, though only one never reported a 10 across the whole study.

### 3.2 Hypoxic apnea intraindividual repeatability

#### 3.2.1 Analysis methods

**FIGURE 3:**
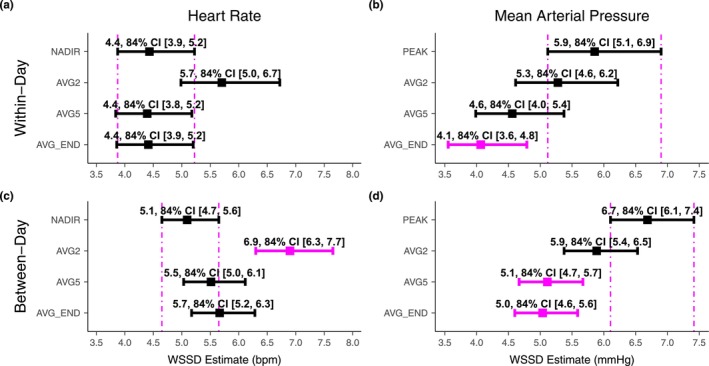
Comparison of the different methods for quantifying within‐day repeatability (a and b) and between‐day repeatability (c and d) for heart rate (a and c) and mean arterial pressure (b and d) (*n* = 16: 8 females, 8 males). The magenta dashed vertical lines show the 84% confidence limits of the NADIR/PEAK within‐subject standard deviation (WSSD); any method whose 84% CI does not overlap with the NADIR/PEAK 84% CI is considered different (*p* < 0.05) and is presented in magenta. Note that the 84% CI is only used for comparison between methods and that the wider 95% CI is presented throughout the manuscript. See text for details on NADIR, PEAK, AVG2, AVG5, and AVG_END.

Using 84% CI overlap, the four methods had no effect on heart rate within‐day repeatability, but the AVG2 method indicated worse between‐day repeatability than the NADIR (see Table 3 and Figure 3). However, the AVG5 and AVG_END methods eliminated the between‐day bias seen with the NADIR and AVG2. For mean arterial pressure, the AVG_END method improved within‐ and between‐day repeatability compared to the PEAK. The AVG5 method also improved between‐ but not within‐day repeatability compared to the PEAK. Mean arterial pressure methods had no effect on between‐day bias. Based on these findings, we present all subsequent heart rate and mean arterial pressure data using the AVG_END.

**TABLE 3:** Intraindividual repeatability data for physiological outcomes.

	Within‐day		Between‐day		Day 1 vs. Day 2 (bias)		
	WSSD	ICC	WSSD	ICC	Bias	*p* Value	Hedges *g*_*av*_
	Value [95% CI]	Value [95% CI]	Value [95% CI]	Value [95% CI]	Value [95% CI]		
Δ HR (bpm)							
NADIR	4.4 [3.7, 5.6]	0.79 [0.62, 0.91]	5.1 [4.5, 5.9]	0.71 [0.54, 0.86]	2.6 [0.6, 4.6]	0.011	0.27
AVG2	5.7 [4.7, 7.2]	0.69 [0.48, 0.86]	6.9 [6.1, 8.0]	0.60 [0.41, 0.79]	2.8 [0.2, 5.3]	0.037	0.24
AVG5	4.4 [3.6, 5.5]	0.77 [0.60, 0.90]	5.5 [4.9, 6.4]	0.71 [0.55, 0.86]	1.7 [−0.4, 3.8]	0.105	0.16
AVG_END	4.4 [3.7, 5.6]	0.80 [0.63, 0.91]	5.7 [5.0, 6.5]	0.73 [0.57, 0.87]	1.7 [−0.4, 3.9]	0.107	0.15
Δ MAP (mmHg)							
PEAK	5.9 [4.9, 7.4]	0.80 [0.64, 0.92]	6.7 [5.9, 7.7]	0.73 [0.57, 0.87]	−1.0 [−4.7, 2.7]	0.596	0.05
AVG2	5.3 [4.4, 6.6]	0.80 [0.64, 0.92]	5.9 [5.2, 6.8]	0.75 [0.60, 0.88]	−0.2 [−2.6, 2.2]	0.856	0.02
AVG5	4.6 [3.8, 5.7]	0.82 [0.67, 0.92]	5.1 [4.5, 5.9]	0.79 [0.65, 0.90]	−0.1 [−2.2, 2.0]	0.952	0.01
AVG_END	4.1 [3.4, 5.1]	0.84 [0.71, 0.93]	5.0 [4.4, 5.8]	0.79 [0.64, 0.90]	0.2 [−1.9, 2.2]	0.856	0.02
Δ SpO_2_ (%)	3.8 [3.2, 4.9]	0.76 [0.57, 0.90]	3.4 [3.0, 4.0]	0.80 [0.66, 0.91]	0.4 [−1.0, 1.7]	0.594	0.05
V̇E (L/min)	1.9 [1.6, 2.4]	0.94 [0.88, 0.98]	3.1 [2.7, 3.5]	0.86 [0.75, 0.94]	−0.5 [−1.8, 0.7]	0.385	0.06

*Note*: Bias refers to the mean difference across the four test apneas between days, with a positive value indicating a lesser bradycardia on day 2 compared to day 1. The *p* value is calculated from the bias. Hedges' g_av_ is an effect size of standardized mean difference. See text for details on NADIR, PEAK, AVG2, AVG5, and AVG_END.

Abbreviations: CI, confidence interval; ICC, intraclass correlation coefficient; V̇E, minute ventilation; WSSD, within‐subject standard deviation; ΔHR, heart rate; ΔMAP, mean arterial blood pressure; ΔSpO_2_, change in peripheral oxygen saturation from free‐breathing to apnea nadir.

#### 3.2.2 Cardiovascular repeatability

**FIGURE 4:**
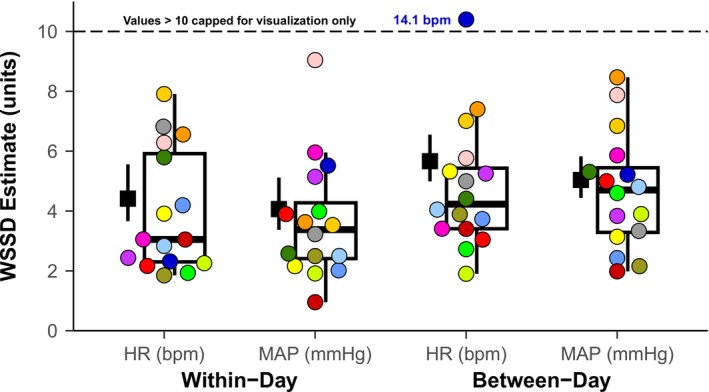
Visual representation of intraindividual repeatability for heart rate (HR) and mean arterial pressure (MAP) using the AVG_END method. The colored dots in all panels show each participant's individual within‐subject standard deviation (WSSD_*i*_), and each color is a distinct participant (*n* = 16: 8 females, 8 males). The boxplots show the median and IQR of WSSD_*i*_. The group WSSD and 95% confidence intervals are shown by the black square and error bar.

The within‐day heart rate AVG_END WSSD was 4.4 bpm, 95% CI [3.7, 5.6] (AVG_END ICC = 0.80, 95% CI [0.63, 0.91]), and the between‐day heart rate AVG_END WSSD was 5.7 bpm, 95% CI [5.0, 6.5] (Figure 4). There was no between‐day bias for heart rate (AVG_END bias = 1.7 bpm, 95% CI [−0.4, 3.9], *p* = 0.107, g_*av*_ = 0.15).

The within‐day mean arterial pressure AVG_END WSSD was 4.1 mmHg, 95% CI [3.4, 5.1] (AVG_END ICC = 0.84, 95% CI [0.71, 0.93]), and the between‐day mean arterial pressure AVG_END WSSD was 5.0 mmHg, 95% CI [4.4, 5.8]. There was no between‐day bias for mean arterial pressure (AVG_END bias = 0.2 mmHg, 95% CI [−1.9, 2.2], *p* = 0.856, g_*av*_ = 0.02). Repeatability for minute ventilation and peripheral oxygen saturation is presented in Table 3.

#### 3.2.3 Outliers

WSSD_*i*_ data revealed statistical outliers in the sample (see Figure 4). Further analysis indicated that one outlier increased the group heart rate WSSD estimates by as much as 24%, and one outlier in mean arterial pressure had a lesser effect (15%). Removing outliers did not change the WSSD comparisons between methods: the AVG_END improved within‐ and between‐day repeatability for mean arterial pressure and there was no difference between heart rate methods. Based on the comparable results all outliers were included in the final analysis. Results with outliers removed are provided in Table S3.

### 3.3 Arrhythmias or ECG changes during hypoxic apneas

Between individuals, arrhythmias or ECG changes had a 45% overall incidence with 14 of 16 participants having at least one arrhythmia or ECG change across their eight test apneas (Table 4). Premature atrial contractions had the highest overall incidence, occurring during 22% of test apneas, followed by junctional escapes at 13%.

**TABLE 4:** Repeatability of arrhythmias or ECG changes during hypoxic apneas using eight test apneas (four test apneas on each day).

Type	Between participants		Within participants	
	Unique participants with arrhythmia (total *n* = 16)	Overall incidence (%)	Affected individuals' incidence (%)	All individuals' incidence (%)
			Median (IQR)	Median (IQR)
All^a^	14	45	44 (25–84)	38 (13–78)
Premature atrial contraction	11	22	25 (13–44)	13 (0–28)
Junctional rhythm	4	13	50 (31–72)	0 (0–3)
Premature ventricular contraction	3	6	13 (13–38)	0 (0–0)
Sinus pause	6	6	13 (13–13)	0 (0–13)
Ectopic atrial rhythm	2	5	38 (38–38)	0 (0–0)
T wave alteration^b^	2	4	31 (22–41)	0 (0–0)

*Note*: Overall incidence is the percentage of apneas with an arrhythmia across all 128 test apneas (16 participants × 8 test apneas). Affected individuals' incidence is the median and interquartile range (IQR) intraindividual arrhythmia incidence, calculated only from participants who had at least one arrhythmia. All individuals' incidence is the median (IQR) intraindividual arrhythmia incidence, including all participants.

^a^ Apneas with multiple arrhythmias or ECG changes were only counted once under “All.” As such, the proportions from the individual types may add up to more than “All.”

^b^ Includes biphasic T waves and nonspecific T wave changes during apneas.

## 4 DISCUSSION

### 4.1 Primary findings

This project aimed to quantify the within‐day and between‐day repeatability of cardiovascular responses to apneas. We also sought to determine how different analysis methods influenced our repeatability metrics. The results largely aligned with our hypotheses. We observed within‐day WSSD estimates of 4.4 bpm and 4.1 mmHg for heart rate and mean arterial pressure, respectively. As expected, between‐day WSSD estimates were higher than within‐day measures. There was no bias between days for heart rate or mean arterial pressure, indicating that random variability likely explains differences between apneas rather than systematic effects. We also observed cardiac arrhythmias or ECG changes during 45% of hypoxic test apneas. However, no specific type of arrhythmia had strong predictive value within or between individuals. Finally, the AVG_END method yielded the best repeatability outcomes when compared to the other analysis methods we tested.

### 4.2 Heart rate and mean arterial pressure

Our findings align with other assessments of heart rate and mean arterial pressure repeatability at rest and during laboratory‐based physiological stressors. A longitudinal study measuring the consistency of resting heart rate and mean arterial pressure noted 5 bpm and 4 mmHg WSSD across two days of testing in 822 individuals (Stanforth et al., 2000). Another group compared responses to static handgrip, post‐exercise circulatory occlusion, and the cold pressor test within and between days in 30 participants (Dillon et al., 2020). At rest, WSSDs were 3–4 bpm and 4–6 mmHg, and they increased to 5–6 bpm and 4–9 mmHg during the physiological stressors. The repeatability of heart rate during cycle ergometer exercise has also been assessed in 19 males, with ~6 bpm WSSD at rest and ~4 bpm WSSD at maximal exercise (Wergel‐Kolmert et al., 2002). Together, these data indicate that under ideal laboratory conditions, cardiovascular variability can be minimized such that the WSSDs approach 5 bpm and 4 mmHg. Some inherent cardiovascular variability remains and must be considered during the design of interventional studies using heart rate or mean arterial pressure as primary outcomes.

A post hoc analysis was unable to identify causes of between‐participant differences in repeatability (i.e., WSSD_*i*_). We saw no relationship between test apnea duration and WSSD_*i*_. In addition, the data were homoscedastic, meaning that the magnitude of change in heart rate or mean arterial pressure during an apnea was not associated with WSSD_*i*_. A recent assessment of cardiovascular repeatability during laboratory‐based physiological stressors noted that a small subset of their participants (2–4 of 30; 7%–13%) had much higher blood pressure variability than the rest of the group (Dillon et al., 2020). Further, outliers were unique between different lab tests and visits, suggesting some randomness not explained by an individual's physiological characteristics. Our outlier data support the observation that random variability in repeated measures can yield stochastic outliers (Dillon et al., 2020).

In the present study, each participant was more like themselves over time compared to others at the same relative timepoint—that is, the WSSD was smaller than the between‐subject standard deviation for a single test apnea. Our ICC estimates confirm this interpretation. This finding indicates nonergodicity; an ergodic process is one that operates the same way in a group of people (e.g., *n =* 20) at one time point as it does in one person over many (e.g., 20) timepoints (Ruissen et al., 2022). Many human processes are nonergodic, and a crucial corollary is that we cannot infer individual‐level responses using group‐level data (Fisher et al., 2018; Speelman & McGann, 2020). Thus, when considering previous data, the finding that high‐altitude hypoxia causes more bradycardia and arrhythmias than normoxia at the group level does not necessarily hold for a randomly selected individual (Busch et al., 2021).

### 4.3 Arrhythmias

The present data corroborate past studies showing transient cardiac arrhythmias occurring during voluntary hypoxic apneas. Across all participants and all test apneas, there was a 45% overall incidence of arrhythmias or ECG changes. Fourteen of 16 participants had at least one arrhythmia during at least one test apnea. Consistent with past research, apnea‐induced changes in cardiac conduction most often presented as ectopic atrial foci (e.g., premature atrial contractions) and junctional escapes (Berthelsen et al., 2022; Busch et al., 2018, 2020, 2021; Kjeld et al., 2021; Lemaître et al., 2013; O'Croinin et al., 2024).

Unlike past studies, our data show that arrhythmia responses are nonergodic and that different arrhythmia types have different group‐to‐individual generalizability. For example, premature atrial contractions were common with 11 of 16 participants exhibiting at least one across their test apneas. However, with only a 25% median affected individuals' incidence, within‐participant consistency was low. On the other hand, junctional rhythms only presented in four participants, but in those participants the median incidence was 50%. The other arrhythmia types were rare between and within participants. Overall, these findings indicate inconsistent and unpredictable apnea responses between participants, within participants, and between arrhythmia types, limiting any generalizability beyond the current sample. Hypoxic apneas cause a high rate of cardiac arrhythmias, but predicting which participants will develop arrhythmias remains a challenge. In the present study, apnea duration did not predict arrhythmia incidence between participants, and consistent durations within participants meant no within‐participant analysis was possible. Currently, we have insufficient data to speculate as to which mechanisms or processes mediate the occurrence of arrhythmias for a given individual during a given apnea.

### 4.4 Analysis methods

As part of our aim to quantify cardiovascular repeatability in response to apneas, we tested whether different analytical methods could alter repeatability independent of protocol standardization. Analysis methods had no meaningful effect on heart rate repeatability, but the AVG_END improved the WSSD estimates for mean arterial pressure. Because the temporal resolution of the different methods affected group mean values for apnea responses (Table 2), we chose the AVG_END for both heart rate and mean arterial pressure. We acknowledge that analysis methods incur trade‐offs; for one, the AVG_END consistently yields smaller group mean change scores than the NADIR/PEAK, which in turn requires a larger sample size to achieve the same statistical power. However, the benefits of repeatable methods outweigh the burden of a small increase in sample size. In addition, the heart rate AVG_END method eliminated the between‐day bias seen with the NADIR method, thus providing a more stable measure of heart rate responses to apneas between days. Lastly, we do not advocate using the AVG2 or AVG5 methods under any circumstances.

Ectopic beats in the final five cardiac cycles of apneas may have caused comparable repeatability between the heart rate NADIR and AVG_END. On the one hand, the AVG_END smooths inherent cardiac variability by averaging five consecutive heart beats. On the other hand, any ectopic beat that shortens the RR interval would skew the AVG_END heart rate but not the NADIR. Indeed, the outlier in heart rate WSSD_*i*_ had repeated premature atrial contractions near the end of their apneas on day 2 that increased heart rate as high as 170 bpm for a single beat. This participant had no premature atrial contractions on day 1, thus only inflating both days' WSSD_*i*_, and presented as an outlier for the AVG_END but not the NADIR. Based on these results we speculate that the AVG_END confers benefits over the NADIR in samples with a low incidence of cardiac arrhythmias (such as in submaximal or normoxic apneas) but that the benefits diminish as ectopic arrhythmias increase (such as in maximal or hypoxic apneas).

### 4.5 Considerations

A past investigation suggests that the cardiovascular system responds differently to apneas after acute hypoxia compared to high‐altitude hypoxia (Busch et al., 2021), including a lower incidence of apnea‐induced arrhythmias and a lesser decrease in heart rate after acute hypoxia. As such, the present results may not accurately represent the repeatability of responses to apneas at altitude. In addition, we did not include any sex differences in this report because we had no reason to believe that one sex would have a more repeatable response to hypoxic apneas than the other. However, we recruited a balanced sample of males and females to ensure generalizability and have reported WSSD and ICC estimates stratified by sex in Table S1. Finally, we standardized end‐tidal partial pressures of carbon dioxide between days starting with the third participant in the study. This participant hyperventilated during their day 1 baseline thus causing 4 mmHg lower end‐tidal carbon dioxide compared to day 2. Following this observation all participants received the same end‐tidal carbon dioxide between days. The first two participants had nearly identical end‐tidal carbon dioxide values between days (<0.5 mmHg and −1.0 mmHg) and were therefore included in the final analysis.

### 4.6 Implications

A growing body of research has indicated that hypoxia augments the cardiovascular response to apneas compared to normoxia, inducing more pronounced bradycardia and more cardiac arrhythmias. However, intraindividual responses may not mimic the interindividual responses previously documented (Fisher et al., 2018). To that end, we do not yet know whether a given response, especially as it relates to arrhythmogenesis, may have prognostic implications. Before we can study the prognostic value of cardiovascular responses to apneas, we must first know the consistency with which the cardiovascular system responds to this stimulus. This study adds to past research by clarifying the repeatability of cardiovascular responses to apneas and showing the lack of repeatability in arrhythmia responses. Future researchers should aim to better understand the triggers of apnea‐induced changes in cardiac conduction to explain why some apneas cause arrhythmias or ECG changes whereas others do not.

## 5 CONCLUSION

In conclusion, we assessed and quantified the within‐day and between‐day repeatability of responses to hypoxic apneas by performing serial apneas on consecutive days in 16 participants. Heart rate and mean arterial pressure responses were robust over time, and analysis techniques using the final five cardiac cycles of the apnea (i.e., AVG_END) displayed the best repeatability. Conversely, arrhythmias and ECG changes showed poor repeatability with large inconsistencies within and between individuals. Together, these data suggest that results from cardiovascular parameters should reproduce, but that researchers should exercise caution in interpreting individual‐level arrhythmia data for prognostic or explanatory purposes. More research is needed to better understand the conditions necessary to cause an arrhythmia or ECG change during an apnea.

## AUTHOR CONTRIBUTIONS

DAY and CDS conceived and designed the research. DAY, BRO, and SvD analyzed the data. DAY, BRO, and MIF performed the experiments. DAY and CDS interpreted the results of the experiments. DAY prepared the figures. DAY drafted the manuscript. DAY, BRO, MIF, SvD, and CDS edited and revised the manuscript. DAY, BRO, MIF, SvD, and CDS approved the final version of the manuscript.

## FUNDING INFORMATION

NSERC Discovery Grant RGPIN‐2020‐05385 (CDS); NSERC CGS‐M (DAY).

## CONFLICT OF INTEREST STATEMENT

No conflicts of interest, financial or otherwise, are declared by the authors.

## ETHICS STATEMENT

The study was approved by the University of Alberta Health Research Ethics Board (Pro00138947) and conformed with the standards of the latest Declaration of Helsinki including registration in a public database (ClinicalTrials.gov NCT06399575).

## Supporting information


Figures S1–S2.

Tables S1–S3.

## Data Availability

Raw data are available on reasonable request to the corresponding author.
