# Supplementary material for: Characterizing the repeatability of cardiovascular responses to hypoxic apneas in adults
Source: Physiol Rep. 2025 Dec 19;13(24):e70692. doi: 10.14814/phy2.70692 (PMC12717465; doi:10.14814/phy2.70692)
Supplement: Supplementary file 1 — Figures S1–S2. [file PHY2-13-e70692-s001.pdf]

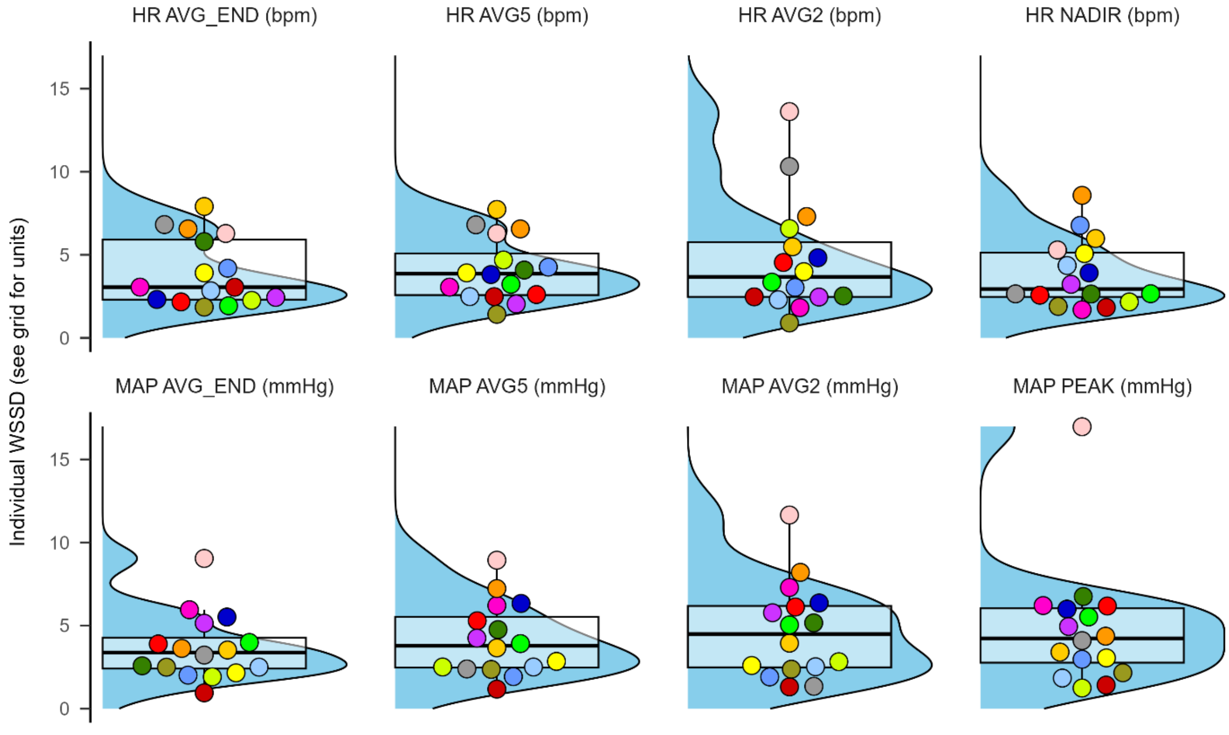

Supplemental Figure S1. Boxplot of individual within-subject standard deviations ($WSSD_i$) for heart rate and mean arterial pressure methods using day 1 data. Any point above the vertical whiskers is 2.5 × interquartile range (IQR) outside the IQR and considered an outlier. For heart rate the dark blue participant is an outlier for both days (see Supplemental Figure S2) and was removed from all heart rate analyses. The light pink participant is an outlier and was removed from all mean arterial pressure analyses.

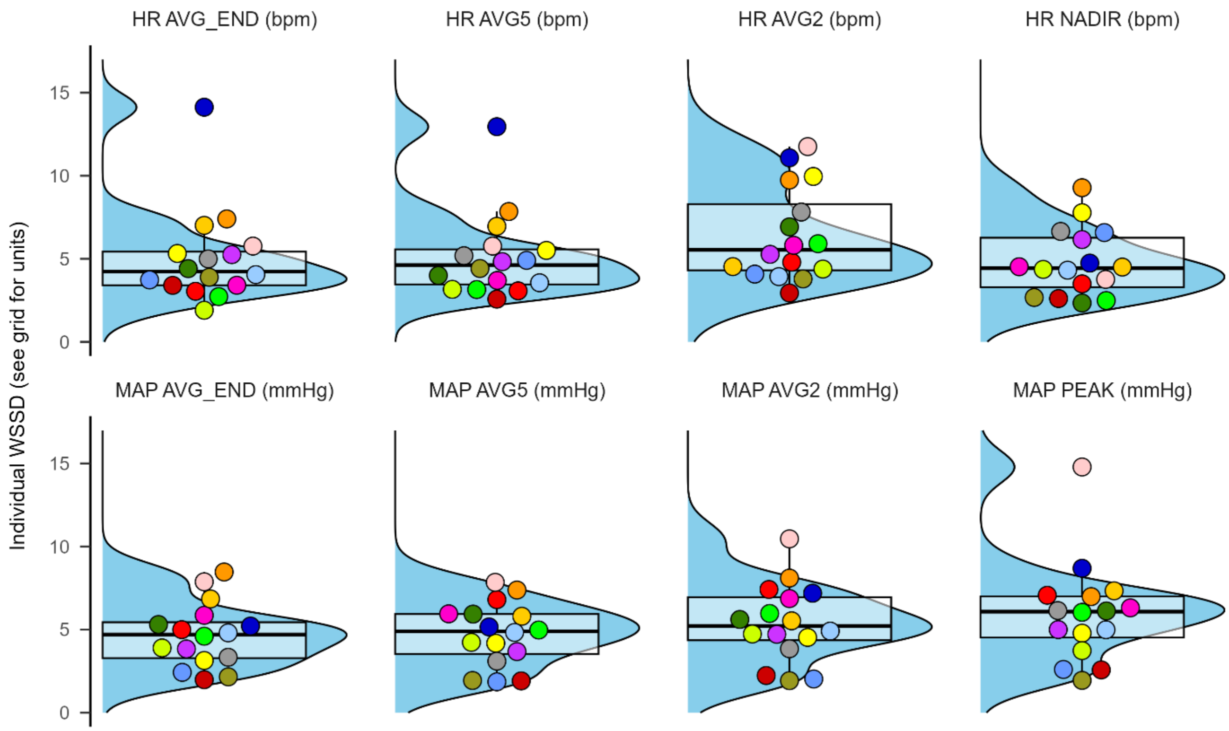

Supplemental Figure S2. Boxplot of individual within-subject standard deviations ($WSSD_i$) for heart rate and mean arterial pressure methods using data from both days combined. Any point above the vertical whiskers is 2.5 × interquartile range (IQR) outside the IQR and considered an outlier. For heart rate the dark blue participant is an outlier for both days and was removed from all heart rate analyses. The light pink participant is an outlier and was removed from all mean arterial pressure analyses.
