# Supplementary material for: Characterizing the repeatability of cardiovascular responses to hypoxic apneas in adults
Source: Physiol Rep. 2025 Dec 19;13(24):e70692. doi: 10.14814/phy2.70692 (PMC12717465; doi:10.14814/phy2.70692)
Supplement: Supplementary file 2 — Tables S1–S3. [file PHY2-13-e70692-s002.pdf]

Supplemental Table S1.
All within-subject standard deviation and intraclass correlation calculations performed as part of this project.
Note. In any instance where a value is rounded more than other data in the same column, the truncated significant digits are all zeros. **WSSD** within-subject standard deviation, **LCL** lower confidence limit, **UCL** upper confidence limit, **ICC** intraclass correlation coefficient, **dbp** diastolic blood pressure, **hr** heart rate, **map** mean arterial pressure, **petco2** end-tidal partial pressure of carbon dioxide, **peto2** end tidal partial pressure of oxygen, **sat** peripheral oxygen saturation, **sbp** systolic blood pressure, **ve** minute ventilation, **delta** change from free-breathing to apnea (see methods), **nadir, max (i.e., peak), avg2**, **avg5**, **avg_end** methods for heart rate and mean arterial pressure (see methods).

Variable	Sex	Day	WSSD	WSSD LCL	WSSD UCL	ICC	ICC LCL	ICC UCL
dbp_delta_avg_end	both	all	4.1	3.6	4.7	3.7	4.5	0.69
dbp_delta_avg_end	both	1	3.5	2.9	4.3	3	4.1	0.72
dbp_delta_avg_end	both	2	4	3.4	5.1	3.5	4.8	0.74
dbp_delta_avg_end	female	all	3.1	2.6	3.9	2.7	3.6	0.56
dbp_delta_avg_end	female	1	2.5	1.9	3.5	2	3.2	0.66
dbp_delta_avg_end	female	2	3.5	2.7	5	2.9	4.5	0.58
dbp_delta_avg_end	male	all	4.7	3.9	5.8	4.1	5.5	0.73
dbp_delta_avg_end	male	1	4.2	3.2	6	3.5	5.4	0.71
dbp_delta_avg_end	male	2	4.6	3.5	6.6	3.8	5.9	0.8
dbp_delta_avg2	both	all	4.8	4.3	5.6	4.4	5.4	0.66
dbp_delta_avg2	both	1	4.2	3.5	5.2	3.6	4.9	0.72
dbp_delta_avg2	both	2	3.8	3.2	4.8	3.3	4.5	0.79
dbp_delta_avg2	female	all	3.5	2.9	4.4	3.1	4.1	0.57
dbp_delta_avg2	female	1	3	2.3	4.3	2.5	3.9	0.68
dbp_delta_avg2	female	2	3.4	2.6	4.9	2.8	4.4	0.66
dbp_delta_avg2	male	all	5.6	4.6	6.9	4.9	6.5	0.68
dbp_delta_avg2	male	1	5.1	3.9	7.3	4.2	6.5	0.69
dbp_delta_avg2	male	2	4.3	3.3	6.2	3.6	5.6	0.84
dbp_delta_avg5	both	all	4	3.6	4.7	3.7	4.5	0.72
dbp_delta_avg5	both	1	3.6	3	4.5	3.2	4.2	0.74
dbp_delta_avg5	both	2	3.6	3	4.5	3.1	4.2	0.8
dbp_delta_avg5	female	all	3.1	2.6	3.9	2.7	3.6	0.61
dbp_delta_avg5	female	1	2.7	2.1	3.9	2.2	3.5	0.67
dbp_delta_avg5	female	2	3.1	2.4	4.5	2.6	4	0.7
dbp_delta_avg5	male	all	4.7	3.9	5.8	4.1	5.5	0.77
dbp_delta_avg5	male	1	4.4	3.4	6.3	3.6	5.7	0.75
dbp_delta_avg5	male	2	4.1	3.1	5.8	3.4	5.3	0.85
dbp_delta_max	both	all	6	5.3	6.9	5.4	6.6	0.61
dbp_delta_max	both	1	4.9	4.1	6.2	4.3	5.8	0.75
dbp_delta_max	both	2	4.4	3.6	5.5	3.8	5.2	0.76
dbp_delta_max	female	all	4.9	4.1	6.1	4.3	5.7	0.55
dbp_delta_max	female	1	3.1	2.4	4.4	2.5	3.9	0.78

dbp_delta_max	female	2	4.8	3.7	6.9	4	6.2	0.66
dbp_delta_max	male	all	6.5	5.5	8.2	5.8	7.7	0.64
dbp_delta_max	male	1	6.4	4.9	9.2	5.3	8.3	0.7
dbp_delta_max	male	2	4.2	3.2	6	3.5	5.4	0.84
hr_delta_avg_end	both	all	5.7	5	6.5	5.2	6.3	0.73
hr_delta_avg_end	both	1	4.4	3.7	5.6	3.9	5.2	0.8
hr_delta_avg_end	both	2	5.8	4.8	7.2	5	6.8	0.76
hr_delta_avg_end	female	all	6.3	5.3	7.9	5.6	7.4	0.65
hr_delta_avg_end	female	1	4.1	3.2	5.9	3.4	5.3	0.78
hr_delta_avg_end	female	2	7.6	5.9	10.9	6.3	9.8	0.62
hr_delta_avg_end	male	all	4.9	4.1	6.1	4.3	5.7	0.79
hr_delta_avg_end	male	1	4.5	3.4	6.4	3.7	5.7	0.82
hr_delta_avg_end	male	2	3.2	2.5	4.6	2.7	4.2	0.91
hr_delta_avg2	both	all	6.9	6.1	8	6.3	7.7	0.6
hr_delta_avg2	both	1	5.7	4.7	7.2	5	6.7	0.69
hr_delta_avg2	both	2	6.1	5.1	7.7	5.4	7.2	0.72
hr_delta_avg2	female	all	6.6	5.5	8.2	5.8	7.7	0.66
hr_delta_avg2	female	1	5	3.8	7.1	4.1	6.4	0.71
hr_delta_avg2	female	2	7.6	5.9	10.9	6.3	9.8	0.65
hr_delta_avg2	male	all	7.2	6	9	6.3	8.4	0.56
hr_delta_avg2	male	1	6	4.6	8.6	5	7.7	0.71
hr_delta_avg2	male	2	4.6	3.5	6.6	3.8	5.9	0.81
hr_delta_avg5	both	all	5.5	4.9	6.4	5	6.1	0.71
hr_delta_avg5	both	1	4.4	3.6	5.5	3.8	5.2	0.77
hr_delta_avg5	both	2	5.5	4.6	7	4.8	6.5	0.76
hr_delta_avg5	female	all	6	5	7.4	5.2	7	0.68
hr_delta_avg5	female	1	4.1	3.2	5.9	3.4	5.3	0.79
hr_delta_avg5	female	2	7.2	5.6	10.3	6	9.3	0.63
hr_delta_avg5	male	all	4.9	4.1	6.2	4.3	5.8	0.74
hr_delta_avg5	male	1	4.3	3.3	6.1	3.5	5.5	0.77
hr_delta_avg5	male	2	3.5	2.7	5	2.9	4.5	0.88
hr_delta_nadir	both	all	5.1	4.5	5.9	4.7	5.6	0.71
hr_delta_nadir	both	1	4.4	3.7	5.6	3.9	5.2	0.79
hr_delta_nadir	both	2	4.2	3.5	5.3	3.6	4.9	0.81
hr_delta_nadir	female	all	4.4	3.7	5.5	3.9	5.1	0.74
hr_delta_nadir	female	1	2.7	2.1	3.9	2.3	3.5	0.91
hr_delta_nadir	female	2	3.9	3	5.6	3.2	5	0.74
hr_delta_nadir	male	all	5.4	4.5	6.8	4.8	6.4	0.69
hr_delta_nadir	male	1	5.7	4.4	8.1	4.7	7.3	0.71
hr_delta_nadir	male	2	4.4	3.4	6.4	3.7	5.7	0.82
hvr	both	all	4.6	4	5.3	4.2	5.1	-0.08
hvr	both	1	0.2	0.2	0.3	0.2	0.3	0.92
hvr	both	2	6.9	5.7	8.7	6	8.1	-0.23
hvr	female	all	6.7	5.5	8.5	5.8	7.9	-0.09
hvr	female	1	0.2	0.2	0.3	0.2	0.3	0.92

hvr	female	2	10	7.6	14.8	8.2	13.2	-0.23
hvr	male	all	0.7	0.6	0.9	0.6	0.8	0.5
hvr	male	1	0.3	0.2	0.4	0.2	0.4	0.92
hvr	male	2	0.6	0.4	0.8	0.5	0.7	0.68
map_delta_avg_end	both	all	5	4.4	5.8	4.6	5.6	0.79
map_delta_avg_end	both	1	4.1	3.4	5.1	3.6	4.8	0.84
map_delta_avg_end	both	2	4.8	4	6	4.2	5.6	0.82
map_delta_avg_end	female	all	4.1	3.4	5.1	3.6	4.7	0.64
map_delta_avg_end	female	1	3.4	2.6	4.8	2.8	4.3	0.72
map_delta_avg_end	female	2	4	3.1	5.8	3.3	5.2	0.71
map_delta_avg_end	male	all	5.6	4.7	7	4.9	6.6	0.83
map_delta_avg_end	male	1	4.7	3.6	6.7	3.9	6.1	0.86
map_delta_avg_end	male	2	5.5	4.3	7.9	4.6	7.1	0.85
map_delta_avg2	both	all	5.9	5.2	6.8	5.4	6.5	0.75
map_delta_avg2	both	1	5.3	4.4	6.6	4.6	6.2	0.8
map_delta_avg2	both	2	4.1	3.4	5.2	3.6	4.8	0.88
map_delta_avg2	female	all	4.8	4	6	4.2	5.6	0.62
map_delta_avg2	female	1	4.2	3.2	6	3.5	5.4	0.7
map_delta_avg2	female	2	4	3.1	5.7	3.3	5.2	0.77
map_delta_avg2	male	all	6.3	5.3	7.9	5.6	7.4	0.79
map_delta_avg2	male	1	6.2	4.7	8.8	5.1	7.9	0.8
map_delta_avg2	male	2	4.2	3.2	6	3.5	5.4	0.92
map_delta_avg5	both	all	5.1	4.5	5.9	4.7	5.7	0.79
map_delta_avg5	both	1	4.6	3.8	5.7	4	5.4	0.82
map_delta_avg5	both	2	3.9	3.2	4.9	3.4	4.6	0.88
map_delta_avg5	female	all	4.3	3.6	5.4	3.8	5.1	0.65
map_delta_avg5	female	1	4.1	3.1	5.8	3.4	5.2	0.68
map_delta_avg5	female	2	4	3.1	5.7	3.3	5.1	0.75
map_delta_avg5	male	all	5.6	4.6	6.9	4.9	6.5	0.83
map_delta_avg5	male	1	5.1	3.9	7.3	4.2	6.6	0.84
map_delta_avg5	male	2	3.9	3	5.6	3.2	5	0.93
map_delta_max	both	all	6.7	5.9	7.7	6.1	7.4	0.73
map_delta_max	both	1	5.9	4.9	7.4	5.1	6.9	0.8
map_delta_max	both	2	4.6	3.8	5.8	4	5.4	0.86
map_delta_max	female	all	5.3	4.4	6.6	4.6	6.2	0.62
map_delta_max	female	1	4	3.1	5.7	3.3	5.1	0.75
map_delta_max	female	2	4.3	3.3	6.2	3.6	5.6	0.78
map_delta_max	male	all	7.4	6.2	9.2	6.5	8.7	0.76
map_delta_max	male	1	7.4	5.7	10.5	6.1	9.5	0.79
map_delta_max	male	2	4.8	3.7	6.9	4	6.2	0.9
petco2	both	all	0.5	0.5	0.6	0.5	0.6	0.98
petco2	both	1	0.3	0.3	0.4	0.3	0.4	0.99
petco2	both	2	0.5	0.4	0.6	0.4	0.6	0.99
petco2	female	all	0.2	0.2	0.3	0.2	0.2	1
petco2	female	1	0.2	0.2	0.3	0.2	0.3	1

petco2	female	2	0.1	0.1	0.2	0.1	0.2	1
petco2	male	all	0.7	0.6	0.9	0.7	0.9	0.96
petco2	male	1	0.4	0.3	0.6	0.3	0.5	0.99
petco2	male	2	0.6	0.5	0.9	0.5	0.8	0.97
peto2	both	all	1	0.8	1.1	0.9	1.1	0.39
peto2	both	1	1	0.8	1.3	0.9	1.2	0.37
peto2	both	2	0.8	0.7	1	0.7	1	0.49
peto2	female	all	0.7	0.6	0.9	0.6	0.8	0.5
peto2	female	1	0.8	0.6	1.1	0.6	1	0.47
peto2	female	2	0.6	0.5	0.9	0.5	0.8	0.44
peto2	male	all	1.2	1	1.5	1	1.4	0.29
peto2	male	1	1.2	1	1.8	1	1.6	0.27
peto2	male	2	0.9	0.7	1.3	0.8	1.2	0.5
sat_delta	both	all	3.4	3	4	3.1	3.8	0.8
sat_delta	both	1	3.8	3.2	4.9	3.3	4.5	0.76
sat_delta	both	2	2.9	2.4	3.7	2.5	3.4	0.84
sat_delta	female	all	3.2	2.6	4	2.8	3.7	0.78
sat_delta	female	1	3	2.3	4.5	2.5	4	0.81
sat_delta	female	2	3.3	2.5	4.9	2.7	4.4	0.72
sat_delta	male	all	3.7	3.1	4.6	3.2	4.3	0.82
sat_delta	male	1	4.6	3.5	6.6	3.8	5.9	0.75
sat_delta	male	2	2.3	1.8	3.3	1.9	2.9	0.9
sat_nadir	both	all	3.1	2.7	3.6	2.8	3.4	0.87
sat_nadir	both	1	3.3	2.7	4.2	2.9	3.9	0.86
sat_nadir	both	2	2.5	2.1	3.1	2.2	2.9	0.91
sat_nadir	female	all	2.8	2.3	3.5	2.5	3.3	0.9
sat_nadir	female	1	2.6	2	3.7	2.1	3.3	0.92
sat_nadir	female	2	2.9	2.2	4.1	2.4	3.7	0.89
sat_nadir	male	all	3.4	2.8	4.2	3	4	0.85
sat_nadir	male	1	4	3.1	5.8	3.4	5.2	0.82
sat_nadir	male	2	2	1.6	2.9	1.7	2.6	0.94
sat_rest	both	all	1.3	1.1	1.5	1.2	1.4	0.71
sat_rest	both	1	1.1	0.9	1.4	1	1.3	0.8
sat_rest	both	2	1.2	1	1.6	1.1	1.5	0.72
sat_rest	female	all	1	0.9	1.3	0.9	1.2	0.65
sat_rest	female	1	0.9	0.7	1.2	0.7	1.1	0.72
sat_rest	female	2	0.8	0.6	1.2	0.7	1	0.8
sat_rest	male	all	1.5	1.2	1.8	1.3	1.7	0.71
sat_rest	male	1	1.3	1	1.9	1.1	1.7	0.81
sat_rest	male	2	1.5	1.2	2.2	1.3	2	0.62
sbp_delta_avg_end	both	all	8	7.1	9.3	7.3	8.9	0.78
sbp_delta_avg_end	both	1	6.2	5.2	7.9	5.5	7.4	0.86
sbp_delta_avg_end	both	2	7.5	6.3	9.5	6.6	8.9	0.81
sbp_delta_avg_end	female	all	6.7	5.6	8.4	5.9	7.8	0.66
sbp_delta_avg_end	female	1	4.8	3.7	6.9	4	6.2	0.79

sbp_delta_avg_end	female	2	6.8	5.2	9.7	5.6	8.7	0.7
sbp_delta_avg_end	male	all	8.6	7.2	10.8	7.6	10.1	0.82
sbp_delta_avg_end	male	1	7.6	5.8	10.8	6.3	9.8	0.86
sbp_delta_avg_end	male	2	8.3	6.4	11.8	6.9	10.7	0.85
sbp_delta_avg2	both	all	8.9	7.8	10.3	8.1	9.9	0.75
sbp_delta_avg2	both	1	7.3	6.1	9.2	6.4	8.6	0.83
sbp_delta_avg2	both	2	7.5	6.2	9.4	6.5	8.8	0.82
sbp_delta_avg2	female	all	7.6	6.4	9.5	6.7	8.9	0.63
sbp_delta_avg2	female	1	6.3	4.9	9.1	5.3	8.2	0.73
sbp_delta_avg2	female	2	7.2	5.5	10.2	5.9	9.2	0.69
sbp_delta_avg2	male	all	9.5	8	11.9	8.4	11.1	0.8
sbp_delta_avg2	male	1	8.5	6.6	12.2	7.1	11	0.84
sbp_delta_avg2	male	2	7.5	5.8	10.8	6.2	9.7	0.88
sbp_delta_avg5	both	all	7.8	6.9	9.1	7.2	8.7	0.78
sbp_delta_avg5	both	1	6.6	5.5	8.3	5.8	7.8	0.84
sbp_delta_avg5	both	2	6.6	5.5	8.3	5.8	7.8	0.84
sbp_delta_avg5	female	all	6.9	5.8	8.6	6.1	8.1	0.66
sbp_delta_avg5	female	1	5.9	4.5	8.4	4.9	7.6	0.74
sbp_delta_avg5	female	2	6.6	5.1	9.4	5.5	8.5	0.71
sbp_delta_avg5	male	all	8.3	6.9	10.4	7.3	9.7	0.83
sbp_delta_avg5	male	1	7.5	5.8	10.8	6.2	9.7	0.87
sbp_delta_avg5	male	2	6.7	5.2	9.6	5.6	8.6	0.9

Supplemental Table S2.

All bias calculations performed as part of this project.

Variable	Bias	Bias UCL	Bias LCL	p Value	Hedges g_{av}
dbp_delta_avg_end	0.6	-1	2.2	0.47	0.08
dbp_delta_avg2	-0.2	-2.2	1.8	0.847	0.02
dbp_delta_avg5	0.3	-1.3	1.9	0.695	0.04
dbp_delta_max	-0.1	-2.5	2.4	0.96	0.01
hr_delta_avg_end	1.7	-0.4	3.9	0.107	0.15
hr_delta_avg2	2.8	0.2	5.3	0.037	0.24
hr_delta_avg5	1.7	-0.4	3.8	0.105	0.16
hr_delta_nadir	2.6	0.6	4.6	0.011	0.27
hvr	0.3	-1.3	1.9	0.74	0.07
map_delta_avg_end	0.2	-1.9	2.2	0.856	0.02
map_delta_avg2	-0.2	-2.6	2.2	0.856	0.02
map_delta_avg5	-0.1	-2.2	2	0.952	0.01
map_delta_max	-0.7	-3.5	2.1	0.628	0.05
sat_delta	0.4	-1	1.7	0.594	0.05
sbp_delta_avg_end	-1.2	-4.5	2	0.449	0.07
sbp_delta_avg2	-1.1	-4.8	2.6	0.546	0.06
sbp_delta_avg5	-1.4	-4.7	1.8	0.376	0.08
sbp_delta_max	-1	-4.7	2.7	0.596	0.05

Note. In any instance where a value is rounded more than other data in the same column, the truncated significant digits are all zeros. **UCL** upper confidence limit, **LCL** lower confidence limit, **Hedges g_{av}** an effect size of standardized mean difference, **dbp** diastolic blood pressure, **hr** heart rate, **map** mean arterial pressure, **sat** peripheral oxygen saturation, **sbp** systolic blood pressure, **ve** minute ventilation, **delta** change from free-breathing to apnea (see methods), **nadir, max (i.e., peak), avg2, avg5, avg_end** methods for heart rate and mean arterial pressure (see methods).

Supplemental Table S3.

Within-subject standard deviation (WSSD) values after removing outliers.

Variable	Day	WSSD	LCL 95%	UCL 95%	LCL 84%	UCL 84%
hr_delta_nadir	all	5	4.4	5.9	4.6	5.6
hr_delta_avg2	all	6.6	5.8	7.7	6	7.3
hr_delta_avg5	all	4.7	4.2	5.5	4.3	5.3
hr_delta_avg_end	all	4.6	4.1	5.4	4.2	5.2
map_delta_max	all	5.8	5.1	6.7	5.3	6.5
map_delta_avg2	all	5.5	4.8	6.3	5	6.1
map_delta_avg5	all	4.9	4.3	5.7	4.4	5.4
map_delta_avg_end	all	4.7	4.2	5.5	4.3	5.3
hr_delta_nadir	1	4.5	3.7	5.7	3.9	5.3
hr_delta_avg2	1	5.8	4.8	7.4	5.1	6.9
hr_delta_avg5	1	4.5	3.7	5.7	3.9	5.3
hr_delta_avg_end	1	4.5	3.7	5.7	3.9	5.3
map_delta_max	1	4.4	3.6	5.6	3.8	5.2
map_delta_avg2	1	4.6	3.8	5.9	4	5.5
map_delta_avg5	1	4.2	3.4	5.3	3.6	4.9
map_delta_avg_end	1	3.5	2.9	4.5	3.1	4.2
hr_delta_nadir	2	4.2	3.5	5.3	3.7	5
hr_delta_avg2	2	5.2	4.3	6.6	4.5	6.1
hr_delta_avg5	2	3.4	2.8	4.4	3	4.1
hr_delta_avg_end	2	3.1	2.5	3.9	2.7	3.7
map_delta_max	2	4.6	3.8	5.9	4	5.5
map_delta_avg2	2	4	3.3	5.1	3.5	4.7
map_delta_avg5	2	3.7	3.1	4.7	3.3	4.4
map_delta_avg_end	2	4.6	3.8	5.9	4	5.5

Note. In any instance where a value is rounded more than other data in the same column, the truncated significant digits are all zeros. **LCL** lower confidence limit, **UCL** upper confidence limit, **95% & 84%** percentage confidence intervals, **hr** heart rate, **map** mean arterial pressure, **delta** change from free-breathing to apnea (see methods), **nadir, max (i.e., peak), avg2, avg5, avg_end** methods for heart rate and mean arterial pressure (see methods).
